# Explainable Survival Modeling in Pediatric Hematopoietic Stem Cell Transplantation Using Landmark‐Based Machine Learning

**DOI:** 10.1155/sci5/7818238

**Published:** 2026-08-10

**Authors:** Khawar Siddiqui, Abdulrahman Al-Musa, Mona Al-Saleh, Hawazen Al Saedi, Abdullah Al-Jefri, Ibrahim Ghemlas, Awatif Al Anazi, Saadiya Khan, Mouhab Ayas, Edward De Vol, Ali Al-Ahmari

**Affiliations:** ^1^ Department of Pediatric Hematology/Oncology, King Faisal Specialist Hospital and Research Center, Riyadh, Saudi Arabia, kfshrc.edu.sa; ^2^ Department of Pediatrics, College of Medicine, Imam Abdulrahman Bin Faisal University, Alkhobar, Saudi Arabia, iau.edu.sa; ^3^ Research Administrative Operations Department, King Faisal Specialist Hospital and Research Center, Riyadh, Saudi Arabia, kfshrc.edu.sa

**Keywords:** acute GvHD, artificial intelligence, black-box model, global explanation, hematopoietic stem cell transplantation, local explanation, pediatric, sinusoidal obstruction syndrome, survex, veno-occlusive disease

## Abstract

**Background:**

Machine learning survival models can outperform conventional Cox regression in heterogeneous pediatric HSCT cohorts, but clinical adoption is limited by concerns regarding information leakage, time‐dependent structure, and interpretability. We evaluated explainable survival modeling aligned to real‐world decision points before transplantation and after early post‐transplant complications.

**Materials and Methods:**

In a single‐center cohort of 839 transplant‐naïve children (< 14 years) undergoing HSCT (2010–2016), overall survival was modeled from infusion to death or last follow‐up. A baseline model used only pretransplant variables (age at HSCT, primary diagnostic group, stem cell source, and donor HLA type). To prevent leakage from postinfusion events, a day + 100 landmark cohort (*n* = 721; 121 deaths beyond day + 100) was constructed and extended with early complications known by day + 100 (SOS/VOD and acute GvHD). Cox proportional hazards, random survival forests (ranger), and randomForestSRC (rfSRC) were benchmarked using C‐index, integrated cumulative/dynamic AUC (iAUC), and integrated Brier score (IBS). Cox proportional hazard assumptions were tested using Schoenfeld residuals. Model‐agnostic explainability (survex) included time‐dependent permutation importance, partial dependence survival profiles, SurvSHAP(t), ceteris paribus profiles, and additive attributions (breakdown and SHAP).

**Results:**

Overall mortality was 28.1% (236/839). Baseline Cox violated proportional hazards (global *p* = 0.00181), whereas the day + 100 landmark Cox did not (global *p* = 0.52). Baseline performance favored ML models (C‐index: Cox 0.673, ranger 0.677, rfSRC 0.668; iAUC: 0.698, 0.866, 0.826; IBS: 0.159, 0.126, 0.135). In the landmark setting, discrimination and accuracy improved with ML (C‐index: Cox 0.721, ranger 0.870, rfSRC 0.840; iAUC: 0.609, 0.668, 0.650; IBS: 0.125, 0.0969, 0.1016). Explainability analyses produced clinically coherent global and individualized risk narratives beyond day + 100.

**Conclusion:**

An information‐leakage‐free, landmark‐based XAI framework allows clinically credible survival prediction in pediatric HSCT, with random survival forests outperforming Cox regression and providing transparent cohort‐level and patient‐level explanations suitable for clinical communication and risk updating.

## 1. Introduction

Despite a multitude of concerns over transparency voiced by stakeholders in oncology and epigenome‐wide landscape of cancer prognosis [1–4], machine learning (ML) continues to evolve and generate interest among hemato‐oncologists and hematopoietic stem cell transplant physicians alike [5, 6]. With the availability of classification models with a variety of techniques to counter challenges related to class‐imbalanced real‐world data, data‐driven ML‐based prediction tools have proven superiority over conventional multivariable statistical models [3, 7–11]. Overall survival (OS) has been and shall continue to serve as a classical endpoint in oncology, stem cell and solid organ transplantation, and cellular therapy research trials [12, 13]. Successful implementation of ML techniques to model survival data as well requires them to be tailored according to the stakeholders’ requirements [14–16]. Limited availability of survival ML algorithms, software to implement them, and the interpretation of the results thus produced are contributing toward its widespread use. The black‐box nature of the ML algorithms, which renders them toward lack of transparency in their decision‐making process, makes it difficult for physicians to understand and trust their recommendations[17]. Recently proposed ML (XAI) algorithms have successfully attempted to address improvement in explainability, transparency, and interpretability of their output, making them acceptable for physicians to incorporate ML recommendations in their routine practice and decision‐making [18–21]. Recently proposed XAI‐based R package named “survex” for ML survival modeling of time‐to‐event data by risk factors with graphical visualizations seems to be a promising approach to meet this objective [22–25]. Using our real‐world institutional data on pediatric hematopoietic stem cell transplantation (HSCT), we evaluated the application of “survex” for 2 ML algorithms ranger (ranger) and random survival forest (randomForestSRC and rfSRC) in comparison with the Cox proportional hazard regression model (coxph). Beyond discrimination, evaluation of predictive models also requires calibration assessment to ensure that predicted survival probabilities correspond to observed clinical outcomes. The aim of this study was to develop explainable ML models for survival prediction after pediatric HSCT using real‐world clinical data. We compared Cox proportional hazards regression with two random survival forest implementations within a framework designed to avoid information leakage incorporating baseline and day + 100 landmark analyses and evaluated model performance using discrimination, calibration, and explainable model interpretation.

## 2. Materials and Methods

This study presents an XAI framework for survival prediction in pediatric HSCT, using real‐world clinical data. The cohort consisted of 839 pediatric transplant‐naïve patients (age < 14 years at transplantation) who underwent HSCT between January 1, 2010, and December 31, 2016, at the Department of Pediatric Hematology/Oncology, King Faisal Specialist Hospital and Research Center, Riyadh, Saudi Arabia. The dataset was derived from institutional transplant databases populated as part of routine clinical care. Institutional Review Board approval was obtained, and due to the retrospective analytical design, a waiver of written informed consent was granted.

The primary endpoint was OS, defined as time from stem cell infusion (day 0) to death from any cause. Patients alive at last follow‐up were censored at the date of last contact. Survival time was calculated in days to allow precise time‐indexed modeling and subsequently expressed in months for reporting purposes (days divided by 30.4375).

To ensure methodological rigor and prevent future information bias, the analytical strategy was structured around two clinically distinct prediction timelines. The first timeline addressed pretransplant risk stratification at the time of infusion. Only variables known at day 0 were included in this baseline model: age at transplant in years (AGESCTYRS), primary diagnostic‐related group (PRIDRG), stem cell source (SOURCEHSC), and donor HLA type (HLATYPE). These variables are fixed at the time of transplant and therefore appropriate for pre‐infusion survival prediction.

The second timeline addressed dynamic prognostic reassessment among patients who survived to day + 100 following transplantation. A landmark cohort was defined by restricting analysis to patients alive and under observation at day + 100. Follow‐up time for landmark models was recalculated from day + 100 until death or last contact. Early post‐transplant complications that occur within the first 100 days were incorporated as predictors in this model. Hepatic sinusoidal obstruction syndrome/veno‐occlusive disease (SOS/VOD) is a life‐threatening disease after allogeneic HSCT, with a high mortality rate, particularly if it is severe [26]. SOS/VOD status (SOS_by_100) was determined using recorded diagnosis dates relative to infusion and coded as “occurred by day + 100: yes/no.” Acute graft‐versus‐host disease (aGvHD) was included as a binary variable reflecting occurrence within the first 100 days (aGvHD) (Table 1). This landmark framework preserves clinical interpretability while avoiding the inappropriate inclusion of post‐transplant events in baseline prediction models.

**TABLE 1:** Patient characteristics and transplant‐related parameters.

Characteristics	Total (*n* = 839)
Recipient gender	
Female	397 (47.3%)
Male	442 (52.7%)
Age at infusion in years (median, range)	3.8 (0.08–17.4)
Primary diagnostic‐related groups	
Malignant disorders	268 (31.9%)
Immune disorders	243 (29.0%)
Hemoglobinopathies	121 (14.4%)
Bone marrow failures	110 (13.1%)
Histiocytic disorders	62 (7.4%)
Metabolic disorders	35 (4.2%)
GvHD prophylaxis	
Negative	151 (18.0%)
Positive	688 (82.0%)
Conditioning regimen	
None	76 (9.1%)
Myeloablative	715 (85.2%)
RIC	48 (5.7%)
Conditioning chemotherapy	
None	76 (9.1%)
Bu ± other agents	87 (10.4%)
Bu/Cy	366 (43.6%)
Others	310 (36.9)
Radiation in conditioning regimen	
TBI‐negative	734 (87.5%)
TBI‐positive	105 (12.5%)
Graft type	
Allogeneic	746 (88.9%)
Autologous	93 (11.1%)
Source of stem cells	
Bone marrow	561 (66.9%)
Cord blood	175 (20.9%)
PBSC	103 (12.3%)
Donor gender	
Female	352 (48.5%)
Male	374 (51.5%)
HLA type (*n* = 746)	
Related matched	518 (69.4%)
Related mismatched 1‐AG	23 (3.1%)
Related haploidentical	33 (4.4%)
Unrelated matched	16 (2.1%)
Unrelated mismatched	156 (20.9%)
SOS/VOD	
Negative	738 (88%)
Positive	101 (12.0%)
Acute GvHD (at day + 100)	
Negative	619 (73.8%)
Positive	220 (26.2%)

*Note:* Values are *n* (%) or median (range) as applicable.

The distinction between time‐dependent covariates and time‐dependent effects was explicitly considered. SOS/VOD represents a covariate whose value changes over follow‐up when represented with diagnosis dates. Rather than modeling it as a continuously time‐varying covariate within a single extended Cox framework, a clinically transparent landmark approach was adopted. This strategy reflects real‐world decision‐making, in which risk reassessment occurs at defined clinical milestones, and avoids structural complexities that may obscure interpretation. Baseline models therefore contain only fixed pretransplant variables, whereas landmark models incorporate early complications known by day + 100.

Three survival modeling approaches were applied independently within each timeline: Cox proportional hazards regression (coxph) as a classical benchmark model, random survival forests implemented via the “ranger” and “randomForestSRC” packages. All models within each timeline used identical predictor sets to ensure comparability. Proportional hazards assumptions for Cox models were evaluated using Schoenfeld residuals via the “cox.zph” function. Where baseline models demonstrated deviation from proportional hazards, coxph was retained as a conservative reference model, while acknowledging that random survival forests do not rely on proportional hazards assumptions.

Model performance was evaluated using time‐dependent discrimination and accuracy metrics. Discrimination was quantified using the concordance index (C‐index) and the cumulative/dynamic area under the receiver operating characteristic curve (AUC) across time. Global discrimination was summarized using the integrated cumulative/dynamic AUC (iAUC). Predictive accuracy was assessed using the Brier score as a time‐dependent measure of prediction error, with overall accuracy summarized using the integrated Brier score (IBS). Time‐dependent AUC and Brier curves were visualized to illustrate model performance across follow‐up, and integrated metrics were used for primary between‐model comparisons. Because Brier scores are inherently time‐indexed and may vary over follow‐up as event rates accumulate, IBS was used as the principal summary of calibration and overall predictive accuracy.

Model calibration was evaluated for the day + 100 landmark models to assess agreement between predicted and observed survival probabilities. Calibration was assessed at a clinically relevant prediction horizon of 365 days following the landmark time point. Predicted risks from each model (Cox proportional hazards, ranger random survival forest, and randomForestSRC survival forest) were grouped and compared with observed event probabilities estimated using inverse probability of censoring weighting (IPCW). Calibration curves were constructed by plotting predicted risk against the corresponding observed risk, with perfect calibration represented by the 45° identity line. This approach provides a direct assessment of whether model‐predicted survival probabilities correspond to empirically observed outcomes.

To enhance interpretability and clinical usability, XAI techniques were applied using the “survex” framework. Global model explanations included permutation‐based variable importance and partial dependence survival profiles. Permutation importance was computed by randomly permuting predictor values and quantifying the resulting change in prediction error, thereby isolating the contribution of each variable to model performance. Local interpretability for individual patients was achieved using survival‐level additive explanations and ceteris paribus survival curves to visualize how predicted survival changes under hypothetical modifications of specific covariates.

All statistical analyses were performed in R (Version 4.2.1) using survival modeling packages for Cox regression, “ranger” and “randomForestSRC” for random survival forests, and “survex” for explainable survival analysis. IBM SPSS Statistics (Version 20.0) was used for supporting descriptive analyses consistent with institutional practice.

### 2.1. Statistical Analysis

The primary outcome of interest, the OS, defined as time in days from infusion of stem cell (day zero) to death from all causes or the date of last follow‐up, was studied against SOS/VOD and other known risk factors recorded at baseline (day 0) and during the first 100 days of transplant in a landmark analysis. Kaplan–Meier curves were drawn for survival analyses. Log‐rank (Mantel–Cox) or Taron‐ware tests were used to test for the significance of the difference between the survival of various groups in a univariate setting. Fisher’s exact test was employed to test for the significance of association between mortality and categorical variables in a univariate setting. All continuous variables were expressed as median (range).

## 3. Results

With a median follow‐up time of 50.7 ± 1.8 months, 5‐year cumulative probability of OS was 70.3% ± 1.7%, which was significantly lower among patients who had SOS/VOD (45.2% ± 05.1%) as compared to the rest of the group (73.7% ± 1.7%, *p* < 0.001). Overall mortality rate for our patients was 28.1% (*n* = 236), which was significantly higher (*n* = 54 of 101, 53.5%) in the SOS‐/VOD‐positive group compared to those children who did not develop SOS/VOD (*n* = 182 of 738, 24.7%) with an odds ratio of 3.510 (95% confidence interval: 2.294–5.370, *p* < 0.001).

**FIGURE 1:**
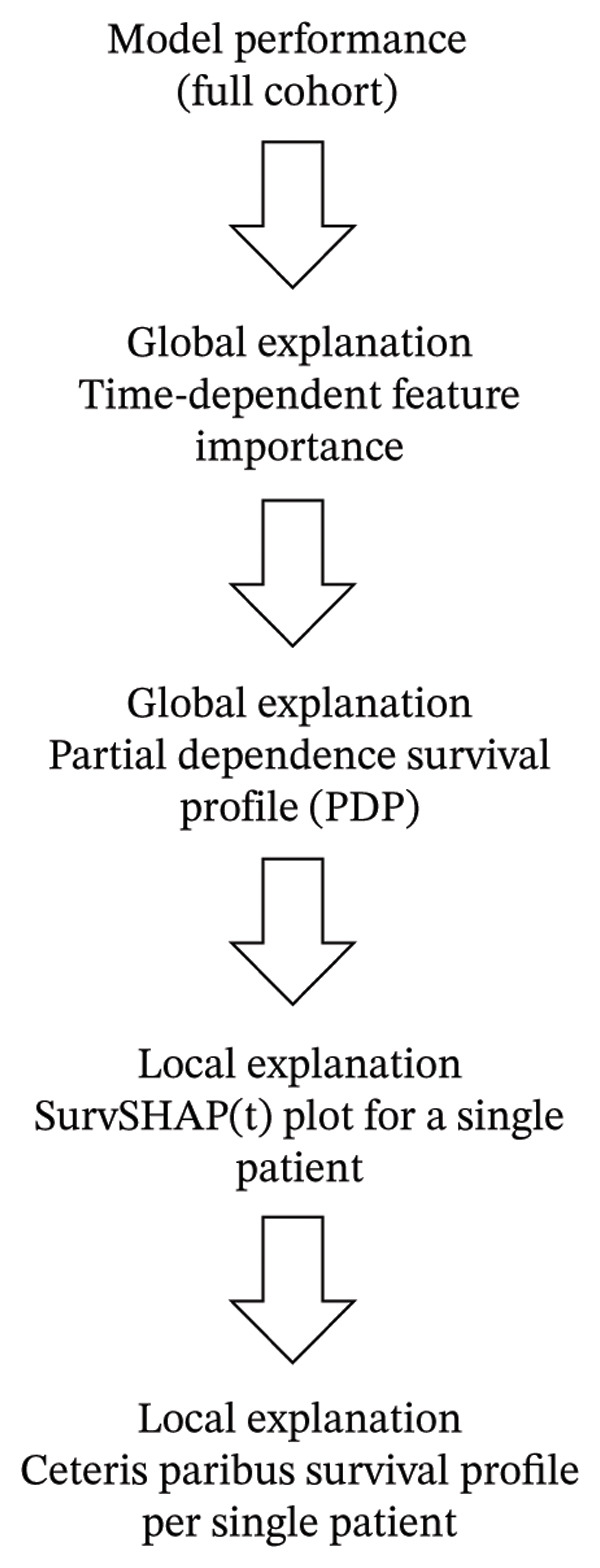
Workflow depicting XAI for survival.

Sequential dedicated and specific workflow was followed for evaluating XAI for survival using the package “survex” for R environment (Figure 1). Model performance estimation for coxph, rfSRC and ranger models, led to the next step of using XAI for OS estimation.

### 3.1. Model Performance

All three models were benchmarked using model quality panel of performance measures namely IBS (the lower, the better), iAUC (the higher, the better), and the C‐index (the higher, the better). OS analysis using unadjusted Cox proportional hazard model is provided in Table 2. At baseline (day 0), using only pretransplant variables (age at transplant, primary diagnostic group, stem cell source, and donor type), all three models demonstrated moderate discrimination. The Cox proportional hazards model yielded a corrected C‐index of 0.673, with an iAUC of 0.599 and an IBS of 0.183. The random survival forest models showed improved performance relative to Cox. The ranger implementation achieved an OOB C‐index of 0.677, iAUC of 0.667, and IBS of 0.164, while the “rfSRC” model demonstrated an OOB C‐index of 0.668, iAUC of 0.686, and IBS of 0.152. These findings indicate that ML‐based survival forests provided superior discrimination and calibration compared with the classical Cox model in the heterogeneous pretransplant setting (Supporting Figures 1 and 2, Table 3).

**TABLE 2:** Overall survival using unadjusted cox proportional hazard model after allogeneic HSCT.

	Univariate model		Multivariable model	
	HR (95 CI)	*p*	HR (95 CI)	*p*
Age	0.951 (0.921–0.981)	0.002	0.993 (0.94–1.033)	0.719
SOS/VOD (positive vs. negative)	2.900 (2.139–3.931)	< 0.001	2.978 (2.145–4.134)	< 0.001
Primary DRG		< 0.001		0.001
Histiocytic disorders vs. malignant disorders	1.128 (0.721–1.763)	0.598	0.633 (0.374–1.072)	0.089
Immune disorders vs. malignant disorders	0.840 (0.620–1.138)	0.259	0.701 (0.472–1.041)	0.078
Metabolic disorders vs. malignant disorders	1.100 (0.617–1.963)	0.747	0.738 (0.396–1.373)	0.337
Bone marrow failures vs. malignant disorders	0.410 (0.248–0.678)	0.001	0.460 (0.270–0.785)	0.004
Hemoglobinopathies vs. malignant disorders	0.217 (0.116–0.405)	< 0.001	0.268 (0.139–0.516)	< 0.001
Source of stem cells		< 0.001		0.205
Cord blood vs. bone marrow	3.208 (2.426–4.242)	< 0.001	1.653 (0.943–2.896)	0.079
PBSC vs. bone marrow	1.780 (1.218–2.601)	0.003	1.058 (0.368–3.041)	0.916
Donor HLA type		< 0.001		0.316
Autologous vs. full‐matched/HLA identical	1.833 (1.232–2.728)	0.003	1.259 (0.410–3.871)	0.687
Others vs. full‐matched/HLA identical	2.854 (2.168–3.758)	< 0.001	1.524 (0.884–2.628)	0.129

**TABLE 3:** Model performance and benchmarking using three models for baseline and landmark analysis.

	C‐index (Cox‐corrected; RSF OOB)	Integrated C/D AUC (iAUC)	Integrated Brier score (IBS)
Baseline timeline (Day 0)			
CoxPH (coxph)	0.673	0.599	0.183
RSF (ranger)	0.677	0.667	0.164
RSF (randomForestSRC)	0.668	0.686	0.152
Landmark analysis (Day + 100)			
CoxPH (coxph)	0.721	0.609	0.125
RSF (ranger)	0.722	0.668	0.0969
RSF (randomForestSRC)	0.719	0.650	0.1016

In the landmark analysis restricted to patients alive at day + 100 (*n* = 721, number of events = 121) and incorporating early post‐transplant complications (SOS/VOD and aGvHD), overall predictive performance improved across models. The Cox model achieved a C‐index of 0.721, with an iAUC of 0.609 and IBS of 0.125, and satisfied proportional hazards assumptions. The ranger‐based random survival forest yielded the highest performance (OOB C‐index 0.722, iAUC 0.668, IBS 0.0969), followed closely by the “rfSRC” implementation (OOB C‐index 0.719, iAUC 0.650, IBS 0.1016). The inclusion of early post‐transplant complications substantially refined risk stratification, and ML models continued to demonstrate superior predictive accuracy compared with Cox, likely reflecting their ability to capture nonlinear interactions in the post‐transplant period (Figures 2(a) and 2(b), Table 3).

FIGURE 2(a) Summary performance metrics for landmark models. Legend: Summary benchmarking of survival models fitted in the day + 100 landmark cohort. Bars compare Cox proportional hazards, ranger random survival forest, and randomForestSRC random survival forest using concordance index (C‐index), integrated cumulative/dynamic AUC (iAUC), and integrated Brier score (IBS). Higher C‐index and iAUC indicate superior discrimination, whereas lower IBS indicates better overall prediction accuracy across the evaluation time grid. Random survival forests outperform Cox regression on all three metrics, with ranger achieving the highest discrimination and lowest integrated prediction error. (b) Time‐resolved model performance after day + 100 landmark. Legend: Time‐dependent prediction performance of Cox proportional hazards (blue), ranger random survival forest (red), and rfSRC random survival forest (green) in the day + 100 landmark cohort (patients alive at day + 100 with follow‐up measured from the landmark). The left panel displays the time‐dependent Brier score, with lower values indicating better predictive accuracy. The right panel displays the cumulative/dynamic area under the ROC curve (C/D AUC), with higher values indicating better discrimination over time. Rug plots along the *x*‐axis denote the distribution of observed event times and censoring during follow‐up. Across most time horizons, both survival forest models demonstrate improved discrimination and lower prediction error compared with Cox regression, with ranger showing the most favorable overall performance.

**Figure:**
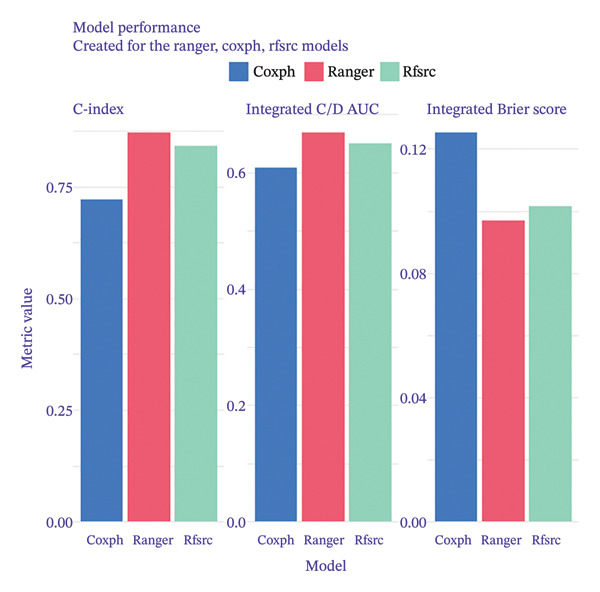
(a)

**Figure:**
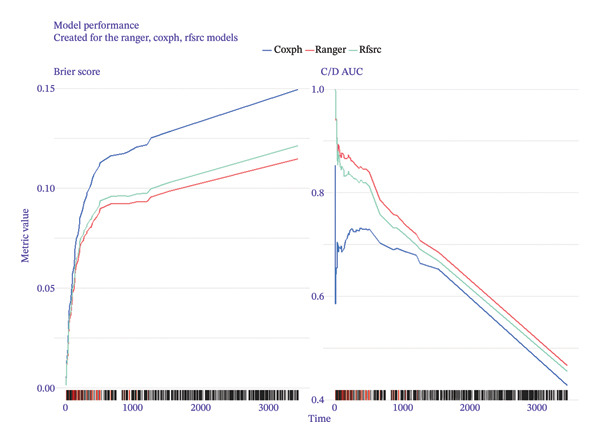
(b)

Calibration assessment at 365 days after the day + 100 landmark demonstrated broadly concordant predicted and observed risks across models, indicating that the improved discrimination achieved by the random survival forest approaches was not accompanied by substantial miscalibration. The random survival forest models showed higher discrimination than the Cox proportional hazards model but exhibited modest overestimation of risk at higher predicted probabilities. The Cox model produced more compressed predictions with comparatively lower risk dispersion. Overall, the ranger random survival forest achieved the highest discrimination while maintaining reasonable calibration, whereas the randomForestSRC implementation showed slightly closer agreement with the ideal calibration line across intermediate risk ranges (Supporting Figure 3).

### 3.2. Global Model Interpretation: Time‐Dependent Variable Importance

Permutation‐based global importance analysis revealed distinct temporal patterns in the determinants of survival. In the baseline (pretransplant) models, stem cell source emerged as the most influential predictor across both random survival forest implementations. Donor HLA type demonstrated the second highest contribution, followed by primary diagnostic category (PRIDRG). Age at transplantation showed comparatively modest influence. The consistency of this ranking across “ranger” and “rfSRC” supports the robustness of this pattern. These findings indicate that, before infusion, procedural and donor‐related characteristics exert the strongest influence on predicted survival, with disease category contributing additional but comparatively smaller predictive value.

**FIGURE 3:**
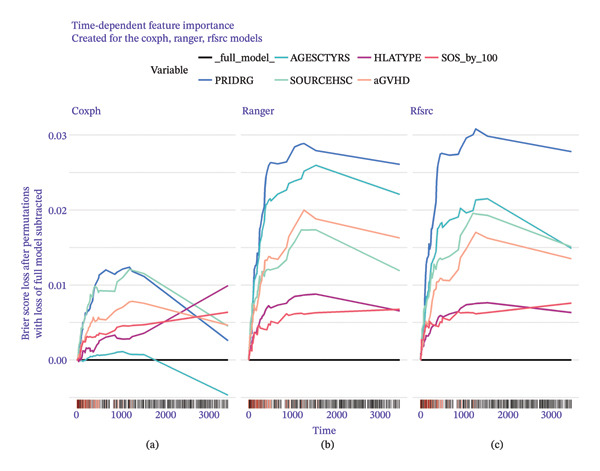
Time‐dependent permutation importance for landmark survival models beyond day + 100. Curves represent the increase in Brier score after variable permutation relative to the full model across follow‐up time. Panels correspond to Cox proportional hazards (a), ranger random survival forest (b), and randomForestSRC (c). Higher values indicate greater contribution to prediction accuracy. Primary diagnostic category and stem cell source demonstrated dominant and sustained influence across models, whereas acute GvHD and SOS/VOD showed earlier and comparatively attenuated effects.

In contrast, the importance structure shifted in the day + 100 landmark models. Time‐dependent permutation importance analysis performed on the landmark cohort demonstrated consistent patterns across the Cox proportional hazards and both random survival forest implementations. Among post–day + 100 predictors, PRIDRG emerged as the dominant determinant of survival, exhibiting the largest increase in Brier score following permutation and maintaining its influence across the duration of follow‐up. Stem cell source represented the second most influential variable, with pronounced early contribution that gradually attenuated over time yet remained substantial throughout the observed horizon. aGvHD displayed moderate early prognostic relevance, peaking within the first 1–2 years beyond the landmark before declining, whereas sinusoidal obstruction syndrome diagnosed within the first 100 days demonstrated comparatively modest but persistent influence. Donor HLA type contributed intermediate prognostic value, while age at transplantation showed minimal time‐dependent impact. Notably, the relative ordering of feature importance was highly concordant between “ranger” and “rfSRC” models, supporting the robustness of these findings and reinforcing the biological plausibility of the survival profile observed beyond day + 100 (Table 4, Figure 3).

**TABLE 4:** Global model interpretation by time‐dependent variable importance for baseline and landmark analysis.

	Baseline timeline (Day 0)		Landmark analysis (day + 100)	
	RSF (ranger)	RSF (randomForestSRC)	RSF (ranger)	RSF (randomForestSRC)
Source of stem cell	0.07235965	0.05806446	0.059986306	0.16010435
Donor HLA type	0.05132964	0.05441317	0.035310512	0.03302226
Primary DRG	0.04380337	0.04134204	0.082312659	0.21625399
Age at transplant (years)	0.01270317	0.01538460	0.022613041	0.06604865
Acute GvHD (at day + 100)	Not applicable	Not applicable	0.041127280	0.11016397
SOS/VOD (at day + 100)	Not applicable	Not applicable	0.004638664	0.05928682

These findings demonstrate that the pattern of mortality risk in pediatric HSCT evolves over time. Before transplantation, survival prediction is driven predominantly by graft source and donor characteristics. After survival to day + 100, intrinsic disease biology and early alloimmune complications assume greater importance, while the relative influence of baseline donor factors attenuates. The temporal reordering of predictor importance underscores the dynamic nature of post‐transplant risk and supports the clinical relevance of a staged prediction framework. The use of time‐dependent global importance measures provides a transparent, clinician‐interpretable representation of how risk profile evolves across the transplant course.

### 3.3. Global Explanation: Partial Dependence Survival Profile

**FIGURE 4:**
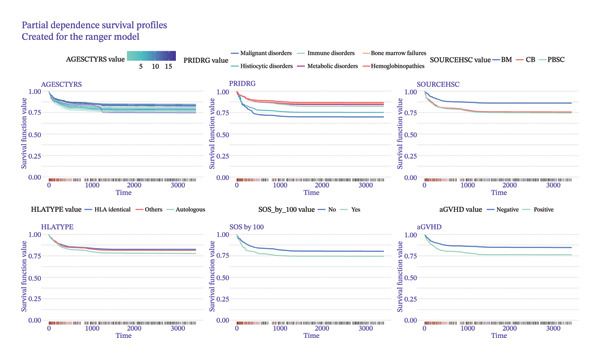
Partial dependence survival profiles from the day + 100 landmark random survival forest (ranger). Legend: Partial dependence survival profiles derived from the ranger random survival forest fitted in the day + 100 landmark cohort. Curves represent the model‐predicted survival function across values or categories of each feature while averaging over the observed distribution of all other covariates. Panels display age at HSCT (AGESCTYRS), primary diagnostic category (PRIDRG), stem cell source (SOURCEHSC), donor HLA type (HLATYPE), SOS/VOD status by day + 100 (SOS_by_100), and acute GvHD status (aGvHD). Separation of curves reflects the relative influence of each feature on model predictions within the landmark framework rather than a causal effect. Distinct shifts in predicted survival are observed across diagnostic groups and graft sources, and the presence of early post‐transplant complications corresponds to lower model‐predicted survival probabilities. Rug plots along the *x*‐axis denote the distribution of follow‐up times and observed events in the landmark cohort.

Partial dependence survival profiles derived from the ranger random survival forest model demonstrated clinically coherent and temporally stable associations between key predictors and post–day + 100 survival. PRIDRG exerted the most pronounced effect, with malignant disorders exhibiting the lowest estimated survival probabilities, whereas hemoglobinopathies and bone marrow failure syndromes demonstrated comparatively favorable trajectories. These separations emerged early and were sustained throughout follow‐up. Stem cell source showed consistent stratification, with bone marrow grafts associated with superior survival compared to cord blood and peripheral blood stem cells. aGvHD and sinusoidal obstruction syndrome diagnosed within the first 100 days were both associated with marked early decrements in predicted survival, with divergence occurring soon after the landmark and persisting over time. Donor HLA type demonstrated modest but discernible influence, while age at transplantation exerted comparatively minimal impact on average survival predictions. Notably, the directionality and relative magnitude of these effects were concordant across both random survival forest implementations, reinforcing the robustness and biological plausibility of the model‐derived associations (Figure 4, Supporting Figure 4).

### 3.4. Local Explanation–SurvSHAP(t) Plot for Single Statistical Unit

**FIGURE 5:**
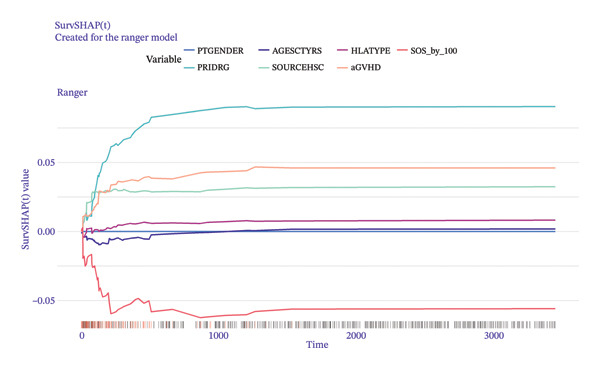
Patient‐level time‐varying feature attribution using SurvSHAP(t) for the day + 100 landmark ranger model (Patient #11). Legend: Time‐varying local feature attributions for Patient #11 estimated using SurvSHAP(t) from the survex package applied to the ranger random survival forest fitted in the day + 100 landmark cohort. Each curve represents the model‐attributed contribution of an individual predictor to the patient’s predicted survival over follow‐up time, displayed on the model’s internal attribution scale; positive and negative values indicate contributions that shift the model prediction in opposing directions relative to the patient‐specific baseline prediction and should not be interpreted as causal effects. Feature contributions vary dynamically across time, with the largest absolute attributions observed for SOS_by_100 status and primary diagnostic category (PRIDRG), followed by stem cell source (SOURCEHSC) and acute GvHD status (aGvHD). In contrast, age at transplantation (AGESCTYRS) and donor HLA type (HLATYPE) demonstrate comparatively modest time‐dependent contributions in this individual profile. Rug marks along the *x*‐axis indicate the distribution of follow‐up times and observed events in the landmark cohort, providing context for regions of greater or lesser empirical support.

Local explanation using SurvSHAP(t) for an individual pediatric recipient (Patient #11; diagnosed with hemoglobinopathy, age at transplant 6.1 years, bone marrow graft from an HLA‐identical donor, SOS/VOD‐positive, no acute GvHD, alive at 3896 days) demonstrated temporally dynamic and clinically coherent contributions of each predictor to the individualized survival trajectory. PRIDRG exerted the strongest positive influence, substantially increasing predicted survival probability relative to the cohort baseline. Bone marrow graft source and absence of aGvHD provided additional favorable contributions, while HLA‐identical donor status conferred a modest protective effect. In contrast, the occurrence of SOS/VOD within the first 100 days imposed a marked early negative contribution to survival prediction, though its impact stabilized over time. Age at transplantation demonstrated minimal influence. Importantly, the aggregate positive contributions outweighed the adverse impact of SOS, consistent with the patient’s observed long‐term survival (Figure 5, Supporting Figure 5). Comparable temporal patterns were observed across both random survival forest implementations, supporting the robustness of individualized explanations. The model attributes survival probability to specific clinical features in a temporally dynamic manner.

### 3.5. Local Explanation–Breakdown Plot and Shapley Additive Explanations

**FIGURE 6:**
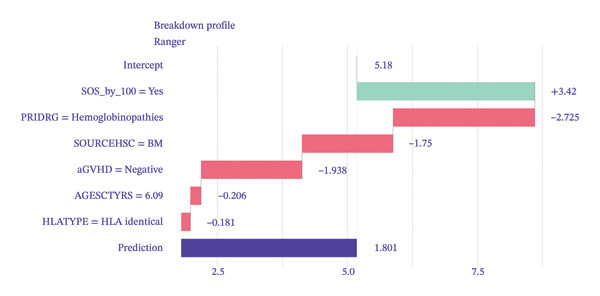
Patient‐level additive attribution (“breakdown”) for the day + 100 landmark ranger model (Patient #11). Legend: Additive patient‐level attribution (“breakdown profile”) for Patient #11 generated using the survex framework from the ranger random survival forest fitted in the day + 100 landmark cohort. The plot decomposes the patient‐specific model prediction into sequential feature contributions starting from an intercept (baseline prediction) and updating the prediction as each feature is incorporated. Bar direction and magnitude indicate how each feature shifts the model prediction relative to the intercept on the model’s internal prediction scale; these contributions summarize model behavior and should not be interpreted as causal effects. In this individual profile, SOS_by_100 status contributes the largest positive shift, whereas PRIDRG category (hemoglobinopathies), stem cell source (bone marrow), and absence of acute GvHD (aGvHD‐negative) contribute negative shifts that partially offset the SOS_by_100 contribution, yielding the final patient‐specific prediction. Age at transplantation (AGESCTYRS) and donor HLA type (HLATYPE) demonstrate comparatively modest contributions in this decomposition. Because breakdown attributions are computed sequentially, the magnitude of individual contributions may depend on the order in which features are introduced and thus reflect one plausible additive explanation of the model prediction rather than a unique decomposition.

Additive local attributions using breakdown decomposition further illustrated how individual predictors contributed to Patient #11’s predicted post–day + 100 survival. Beginning from the cohort‐level baseline prediction, sinusoidal obstruction syndrome within the first 100 days exerted the largest adverse shift in predicted outcome. In contrast, hemoglobinopathy diagnosis produced a substantial protective contribution, partially offsetting the detrimental impact of SOS. Bone marrow graft source and absence of aGvHD provided additional protective shifts, while age at transplantation and HLA‐identical donor status contributed only minimal adjustments to the individualized prediction. The net effect of these additive contributions resulted in a favorable OS estimate, consistent with the patient’s observed long‐term survival (Figure 6). Similar attribution patterns were observed across both random survival forest implementations, supporting the robustness of patient‐level explanations (Supporting Figure 6).

**FIGURE 7:**
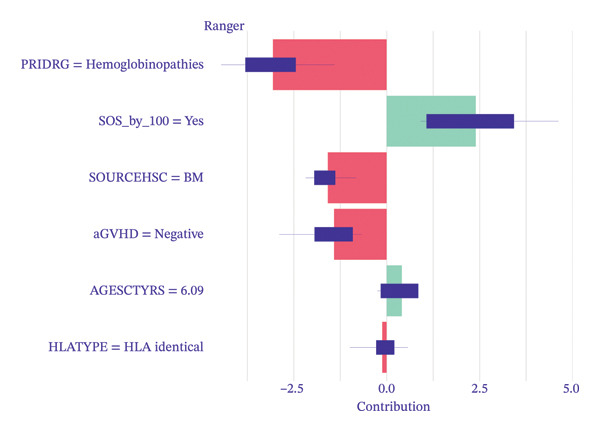
Patient‐level SHAP feature attributions for the day + 100 landmark ranger model (Patient #11). Legend: Patient‐level SHAP (Shapley additive explanations) attribution profile for Patient #11 derived from the ranger random survival forest fitted in the day + 100 landmark cohort. The plot summarizes how each feature contributes to the patient‐specific model prediction relative to a reference (the model’s average prediction), with contributions shown on the model’s internal prediction scale. Positive contributions shift the model prediction in one direction relative to the reference, whereas negative contributions shift it in the opposite direction; these values reflect model behavior and should not be interpreted as causal effects. For this patient, SOS_by_100 status is the dominant positive contributor, while PRIDRG (hemoglobinopathies), aGvHD status (negative), and stem cell source (bone marrow) contribute in the opposite direction, partially offsetting the SOS_by_100 contribution; age at transplantation (AGESCTYRS) and donor HLA type (HLATYPE) provide comparatively smaller contributions. Unlike breakdown decompositions, SHAP attributions are order‐invariant, providing a consistent allocation of contributions across predictors under the Shapley framework.

Local feature attribution using Shapley additive explanations (SHAP) further quantified the contribution of individual predictors to Patient #11’s post–day + 100 survival estimate. Within the ranger random survival forest model, sinusoidal obstruction syndrome demonstrated the largest adverse contribution to the predicted outcome, shifting the individualized estimate toward higher risk. In contrast, hemoglobinopathy diagnosis exerted the strongest protective contribution, substantially offsetting the detrimental effect of SOS. The absence of aGvHD and use of bone marrow graft source provided additional protective contributions of moderate magnitude, whereas age at transplantation and HLA‐identical donor status contributed only minimal adjustments to the individualized prediction. The direction and relative ranking of feature contributions were concordant across both random survival forest implementations, underscoring the stability of local explanations. Collectively, these findings illustrate how opposing clinical factors interact within the model to produce an individualized survival estimate consistent with the patient’s long‐term observed outcome (Figure 7, Supporting Figure 7).

Two complementary local explanation techniques were used to ensure interpretability robustness. Breakdown decomposition illustrates how the prediction shifts from the average baseline to the individualized estimate through sequential additive contributions, whereas SHAP provides an order‐independent, game‐theoretic attribution of feature effects. Presenting both approaches allows intuitive visualization of prediction shifts while confirming that feature contributions remain consistent under a theoretically rigorous attribution framework.

### 3.6. Local Explanation: Instance‐Level Profiles as Ceteris Paribus for Survival

**FIGURE 8:**
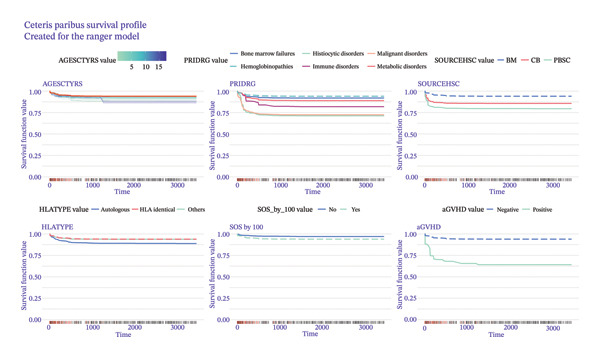
Patient‐level ceteris paribus survival profiles from the day + 100 landmark ranger model (Patient #11). Legend: Ceteris paribus survival profiles for Patient #11 generated using the survex framework from the ranger random survival forest fitted in the day + 100 landmark cohort. Each panel shows the patient’s predicted survival function when a single feature is perturbed across plausible values or categories, while all other patient characteristics are held fixed at their observed values. This visualization therefore represents a local patient‐level sensitivity analysis of the fitted model and should not be interpreted as a causal effect of changing any variable. In this individual, varying PRIDRG and stem cell source (SOURCEHSC) produces discernible shifts in the predicted survival trajectory, while toggling SOS_by_100 and aGvHD status results in marked separation of survival curves, consistent with substantial model sensitivity to early post‐transplant complication status in the landmark setting. Age at transplantation (AGESCTYRS) and donor HLA type (HLATYPE) demonstrate comparatively smaller changes in the patient‐specific survival profile. Rug marks along the *x*‐axis indicate the distribution of follow‐up times and events in the landmark cohort, providing context for where model predictions are most supported by observed data.

Ceteris paribus survival profiles were used to generate counterfactual, patient‐specific explanations for Patient #11. Across both random survival forest implementations, altering PRIDRG produced the largest shifts in individualized survival projections, with hemoglobinopathy consistently associated with the most favorable predicted trajectory and malignant/histiocytic categories yielding lower predicted survival curves. Changing stem cell source also produced persistent separation of survival curves, with bone marrow demonstrating a more favorable predicted trajectory compared with cord blood and peripheral blood stem cells. Among dynamic post‐transplant factors, aGvHD positivity generated the largest counterfactual decrement in survival probability, while SOS/VOD positivity produced a consistent but comparatively smaller reduction. Donor HLA type and age at transplantation resulted in only modest changes in predicted survival when varied counterfactually (Figure 8, Supporting Figure 8). These patient‐level profiles complement global explainability by illustrating how clinically plausible modifications of individual predictors shift the model’s survival estimate while holding other recipient characteristics constant.

## 4. Discussion

Time‐to‐event modeling remains central to pediatric HSCT, where outcomes unfold across heterogeneous diagnostic indications and are influenced by both pretransplant factors and early post‐transplant complications. In parallel, the maturation of ML survival methods has expanded the modeling toolkit beyond classical proportional hazards frameworks, with growing interest in whether these approaches can improve discrimination while preserving clinical interpretability and trust [1, 3, 14]. The present work addresses this translational gap by evaluating two random survival forest implementations alongside Cox proportional hazards modeling and by applying a unified explainability workflow to generate global‐ and patient‐level explanations that are accessible to clinicians. Critically, the analysis was structured to mirror real‐world decision‐making: a pre‐HSCT baseline model intended for counseling and planning at the time of infusion, and a post–day + 100 landmark model intended for dynamic prognostic updating once early complications are known.

A central methodological contribution of this study is the explicit separation of baseline prediction from dynamic post‐transplant updating to avoid information leakage. Early toxicities, such as SOS/VOD and aGvHD, occur after infusion and therefore cannot validly inform predictions made at day 0. Rather than treating these complications as static baseline covariates, we incorporated them within a landmark framework, where only patients alive and under follow‐up at the landmark time contribute to subsequent risk sets, and complication status is defined using information available by that point. This structure aligns the modeling task with the clinical question being asked, and it avoids the invalid use of future information that can otherwise inflate apparent performance. The landmark design also accommodates the practical constraints of retrospective datasets in which exact time stamps for all time‐varying events may be incomplete, while still producing clinically meaningful dynamic risk estimates.

Across both timelines, the ML models demonstrated strong performance relative to the Cox benchmark, with superior discrimination and lower prediction error, consistent with prior reports that ensemble survival methods can capture nonlinearities and interactions that are difficult to prespecify in parametric frameworks [15]. In the baseline setting, performance differences were accompanied by a clinically coherent pattern of global importance, where graft source and donor type tended to dominate, followed by underlying diagnostic category, while age contributed comparatively less. In the post–day + 100 landmark setting, the relative contribution of early complications became more prominent, reflecting the biological reality that early endothelial injury syndromes and alloimmune toxicity shape later survival trajectories after initial transplant recovery [5, 26]. Importantly, although the Cox model served as a familiar reference point for clinicians, proportional hazards assumptions were not uniformly satisfied in the baseline analysis, underscoring the value of complementing classical regression with more flexible ML survival approaches in heterogeneous transplant cohorts.

In addition to discrimination, we evaluated calibration of model predictions at a clinically relevant horizon of one year following the day + 100 landmark. Calibration assessment demonstrated that the survival forest models achieved superior discrimination while maintaining acceptable calibration, although some overestimation of risk was observed at the highest predicted risk levels. This pattern is consistent with prior reports that ensemble survival models may achieve stronger discrimination but require careful calibration assessment [27, 28]. Importantly, the Cox proportional hazards model produced smoother but less discriminative predictions, highlighting the trade‐off between predictive accuracy and calibration observed in survival modeling. To ensure a comprehensive evaluation of predictive performance, we assessed both discrimination and calibration of the models, demonstrating that the improved discrimination achieved by the survival forest approaches was accompanied by acceptable agreement between predicted and observed risks. These findings underscore the importance of reporting both discrimination and calibration metrics when evaluating ML models for clinical prognostication.

The explainability analyses provide a second key contribution by translating model behavior into interpretable clinical narratives. Global explanations, such as time‐dependent permutation importance and partial dependence survival profiles, clarified how, on average, predicted survival changes across key transplant strata, and they highlighted which covariates the models relied on most strongly over follow‐up. Although permutation‐based summaries are often described as “time‐dependent,” they should be interpreted cautiously as reflecting changes in predictive utility across time horizons rather than implying true time‐varying coefficients in the proportional hazards sense. This distinction is particularly relevant when clinicians and reviewers conflate time‐dependent covariates (variables that change value over time) with time‐dependent effects (coefficients that change over time). In our study, baseline covariates were fixed by design; dynamic predictors were introduced only at the landmark, where they represent state variables known by day + 100 rather than continuously updated time‐dependent covariates. Within this framing, changes in permutation‐based importance across time most plausibly reflect the evolving difficulty of prediction and the changing information content of baseline versus early‐complication variables as follow‐up accrues.

Patient‐level explanations further demonstrated how the modeling framework can support individualized clinical reasoning. For a representative long‐term survivor, local methods—including SurvSHAP(t), ceteris paribus survival profiles, and additive attribution (breakdown and SHAP)—consistently showed that adverse contributions from SOS/VOD were counterbalanced by favorable diagnosis group and graft characteristics, yielding an OS estimate concordant with the observed outcome. Presenting both breakdown and SHAP provides complementary perspectives: Breakdown offers an intuitive sequential decomposition that many clinicians find readable, whereas SHAP provides an order‐invariant, game‐theoretic allocation of contributions that is increasingly regarded as a rigorous standard in XAI [18]. The convergence of these independent explanation families is itself informative, because concordant attributions across methods and across two forest implementations increase confidence that the explanations reflect stable model structure rather than artifacts of a single algorithm.

Several limitations merit emphasis. First, this is a single‐center cohort, and external validation will be essential before any clinical deployment. Second, the landmark framework was used to align modeling with available information and to prevent leakage, but it does not replace full time‐dependent covariate modeling; where complete event‐time data are available, future work could extend these analyses to models that represent SOS/VOD and aGvHD as true time‐dependent covariates. Third, local explanation methods remain model‐based summaries rather than causal estimates, and they should be interpreted as describing how the model distributes prediction contributions across features, not as evidence that a given factor “caused” a patient’s outcome. Finally, while integrated metrics, such as iAUC and IBS, provide robust summary performance, they can mask time‐specific calibration issues; complementary reporting of time‐resolved curves and clinically chosen horizons remains important for clinical interpretation.

Despite these constraints, the corrected two‐stage framework advances a pragmatic path for clinically grounded XAI in pediatric HSCT. Baseline models support pre‐infusion counseling and planning using only information available at transplant, while landmark models provide dynamic prognostic updating once early post‐transplant complications have declared themselves. Within each stage, model‐agnostic explainability offers a transparent bridge between predictive performance and clinician trust, enabling transplant teams to interrogate cohort‐level drivers of risk and to examine patient‐level predictions in a manner consistent with clinical reasoning. As ML applications continue to expand across transplant medicine [3, 14], such information‐leakage‐free designs paired with interpretable outputs may help ensure that predictive tools are not only accurate but also clinically interpretable and suitable for implementation. Importantly, model evaluation incorporated both discrimination and calibration assessments, confirming that the enhanced predictive performance of the survival forest models was not achieved at the expense of meaningful miscalibration. Taken together, these findings suggest that ML‐based survival models incorporating early post‐transplant clinical events may provide a more refined framework for dynamic risk stratification following HSCT. Explainable survival modeling may enhance clinical risk assessment after transplantation.

### 4.1. Limitations

Several limitations should be acknowledged. The study was conducted using data from a single transplant center, and therefore, external validation in independent cohorts will be important to confirm the generalizability of the findings. In addition, although the modeling framework incorporated clinically meaningful predictors and rigorous evaluation of discrimination and calibration, future work may explore the integration of additional biological or longitudinal variables to further refine risk prediction.

**Nomenclature**
HSCT: hematopoietic stem cell transplantation.
GvHD: graft‐versus‐host disease.
HLA: human leukocyte antigens.
OS: overall survival.
SOS/VOD: sinusoidal obstruction syndrome, veno‐occlusive disease.

## Author Contributions

All authors certify that they have participated sufficiently in the intellectual content and the analysis of data. Khawar Siddiqui conceptualized the research and carried out formal analysis with Edward De Vol. Mona Al‐Saleh curated and checked the data. Ali Al‐Ahmari, Abdulrahman Al‐Musa, Abdullah Al‐Jefri, Hawazen Al Saedi, Ibrahim Ghemlas, Awatif Al Anazi, Edward De Vol, Saadiya Khan, and Mouhab Ayas supervised the study and contributed toward the write‐up and medical opinions.

## Funding

This work did not receive any financial support in any form from any funding agency.

## Disclosure

All authors reviewed the final version of the manuscript and approved it for publication.

## Ethics Statement

This study was submitted to the Institutional Review Board of King Faisal Specialist Hospital and Research Center, Riyadh, Saudi Arabia, before initiation and was approved by the Research Advisory Committee through established procedures via Approval Number 2171112.

This study was conducted in compliance with the ethical standards of the responsible institution on human subjects as well as with the Helsinki Declaration.

## Consent

Data of interest collected from the patients’ medical records were secured as governed by the institutional policies on patient confidentiality and privacy. No informed consents were obtained as this was a retrospective study, and all data items collected were already documented in medical charts as part of the patient care and disease management.

## Conflicts of Interest

The authors declare no conflicts of interest.

## Supporting Information

Additional supporting information can be found online in the Supporting Information section.

## Supporting information


**Supporting Information 1** Supporting Figure 1 Summary performance metrics for baseline model.

**Supporting Information 2** Supporting Figure 2 Time‐resolved model performance baseline model.

**Supporting Information 3** Supporting Figure 3 Calibration of survival predictions at 365 days following the day + 100 landmark. Legend: Calibration curves comparing predicted versus observed risk at 365 days after the day + 100 landmark for the Cox proportional hazards model, the random survival forest implemented via ranger, and the random survival forest implemented via randomForestSRC. Predicted risks were grouped and compared with observed event probabilities estimated using inverse probability of censoring weighting (IPCW). The diagonal grey line represents perfect calibration. The random survival forest models demonstrated improved discrimination relative to the Cox model while maintaining acceptable calibration across most of the risk spectrum, although modest overestimation was observed at higher predicted risk levels. AUC and Brier score estimates with corresponding confidence intervals are shown for reference.

**Supporting Information 4** Supporting Figure 4 Partial dependence survival profiles from the day + 100 landmark random survival forest (‘rfSRC’).

**Supporting Information 5** Supporting Figure 5 Patient‐level time‐varying feature attribution using SurvSHAP(t) for the day + 100 landmark rfSRC model (Patient #11).

**Supporting Information 6** Supporting Figure 6 Patient‐level additive attribution (“breakdown”) for the day + 100 landmark rfSRC model (Patient #11).

**Supporting Information 7** Supporting Figure 7 Patient‐level SHAP feature attributions for the day + 100 landmark rfSRC model (Patient #11).

**Supporting Information 8** Supporting Figure 8 Patient‐level ceteris paribus survival profiles from the day + 100 landmark rfSRC model (Patient #11).

## Data Availability

The raw data cannot be provided due to institutional restrictions.
